# Integrating artificial intelligence and conventional approaches in sugarcane bagasse biorefineries: a review towards a circular bioeconomy

**DOI:** 10.3389/fbioe.2026.1879932

**Published:** 2026-06-17

**Authors:** Farrag F. B. Abu-Ellail, Ebtehag A. E. Sakr, Tanweer Kumar, Shaimaa A. Nour, Rasha G. Salim, Ghada M. El-Sayed, Peifang Zhao, Chao-Hua Xu, Hongbo Liu, Zhineng Wang, Li Ping Zhao, Xiongmei Ying

**Affiliations:** 1 State Key Laboratory for Tropical Crop Breeding, Sugarcane Research Institute, Yunnan Academy of Agricultural Sciences, Yunnan Key Laboratory of Sugarcane Genetic Improvement, Kaiyuan, China; 2 Breeding and Genetics Department, Sugar Crops Research Institute, Agricultural Research Center, Giza, Egypt; 3 Botany Department, Faculty of Women for Arts, Science, and Education, Ain Shams University, Cairo, Egypt; 4 Sugar Crops Research Institute (SCRI), Mardan, Agriculture Research Department, Khyber Pakhtunkhwa, Pakistan; 5 Chemistry of Natural and Microbial Products Department, Pharmaceutical and Drug Industries Institute, National Research Centre (NRC), Dokki, Egypt; 6 Microbial Genetics Department, Biotechnology Research Institute, National Research Centre (NRC), Dokki, Egypt

**Keywords:** artificial intelligence, biomass valorization, circular bioeconomy, machine learning, sugarcane bagasse, sustainable agriculture

## Abstract

Sugarcane bagasse (SCB), with global production exceeding 200 million metric tons per year, has emerged as a key lignocellulosic feedstock at the heart of the circular bioeconomy, offering a sustainable, low-carbon alternative to fossil resources through production of biofuels, biochemicals, biopolymers, and bioelectricity. This review critically examines the interplay between conventional bioprocess approaches and artificial intelligence (AI) methods in SCB-based biorefineries. It highlights that while traditional thermochemical and biochemical routes provide the physicochemical backbone of value-added processes, these conventional routes face inherent limitations in energy efficiency, operational flexibility, and environmental performance due to high pretreatment costs and biomass recalcitrance. The integration of machine learning (ML) techniques, including artificial neural networks, support vector machines, genetic algorithms, adaptive neuro-fuzzy systems, and digital twins, enables data-driven modeling, real-time process control, predictive maintenance, and multi-objective optimization across SCB pretreatment, hydrolysis, fermentation, and cogeneration units. This integration enhances resource use efficiency, product diversification, and closed-loop material flows, thereby reducing waste and advancing zero-waste circularity. The review underscores the synergistic potential of combining AI with established bioprocess knowledge to advance integrated, scalable SCB biorefineries aligned with circular bioeconomy principles. It also identifies key techno-economic, regulatory, and scalability barriers, including costly pretreatment and feedstock recalcitrance, and proposes coordinated research and policy strategies to accelerate the global deployment of sustainable, bio-based industrial systems grounded in SCB valorization.

## 1 Introduction

Fossil fuel reserves are finite, and their unrestrained use causes severe environmental pollution (Li et al., 2023). At the same time, growing demand for clean, renewable energy has heightened the recognition of agricultural waste as a sustainable feedstock (Periyasamy et al., 2023). Worldwide, agricultural residues reach approximately 998 million metric tons (MT) annually, including rice husks (100–120 MT), wheat straw (400–529 MT), sugarcane bagasse (SCB) (279–300 MT), and corncob waste (200–230 MT) (Velusamy et al., 2022). Sugarcane (*Saccharum spp*.) is a major tropical crop cultivated in over 100 countries (Wani et al., 2023). Its processing generates several byproducts: press mud, molasses, cane waste, and notably bagasse (Iwuozor et al., 2022). SCB, the fibrous residue after juice extraction (Antunes et al., 2022), consists of 45%–50% cellulose, 25%–30% hemicellulose, 17%–25% lignin, and 2.4%–9% ash ((Niju and Swathika, 2019). Global SCB output exceeds 100 MT per year, with a waste rate of 25%–30% resulting in 125–150 MT of residues annually (Vaish et al., 2022). More broadly, agri-waste (crop residues, manure, and agro-industrial by-products) exceeds five billion MT annually and is often mismanaged through open burning, underscoring the need for circular bioeconomy strategies (Mehdizadeh et al., 2025). Although thermochemical and biochemical conversion can turn these feedstocks into biofuels and chemicals, scalability is limited by feedstock variability, high pretreatment costs, and policy gaps.

Consequently, SCB has become a strategic lignocellulosic feedstock for circular bioeconomy biorefineries (Kamboj et al., 2024). Traditionally burned for steam and electricity, modern biorefineries instead convert SCB into biochemicals, biofuels, and biomaterials, enabling cascading biomass use, industrial symbiosis, and closed-loop material flows. Yet techno-economic, social, and policy barriers hinder large-scale deployment ((Ubando et al., 2020). Conventional valorization relies on thermochemical (combustion, gasification, and pyrolysis) and biochemical (anaerobic digestion, fermentation) routes, typically optimized by empirical trial and error. These methods suffer from low energy efficiency, outdated equipment, high feedstock moisture, and inflexible centralized systems, all of which reduce cogeneration performance, limit grid electricity export, slow process optimization, increase emissions, and prevent integration into data-driven circular bioeconomy models (da Silva Aires et al., 2025). In contrast, SCB pretreatment enables production of value-added bioproducts and biofuels, turning problematic waste into a strategic resource (Kamboj et al., 2024).

Researchers are increasingly embedding artificial intelligence (AI) and machine learning (ML) into lignocellulosic biorefining, including SCB-based systems, to accelerate innovation and improve operational efficiency, thereby addressing the aforementioned limitations. ML and optimization models, such as artificial neural networks (ANN), enable data-driven control and parameter tuning for key unit operations (anaerobic digestion, gasification, pyrolysis, enzymatic hydrolysis, briquetting), thereby boosting energy yields and lowering emissions. AI-driven approaches also strengthen predictive modelling, real-time optimization, and digital twin supervision, facilitating intelligent coupling with fuel cells, hybrid power configurations, and energy trading platforms (Garg et al., 2025). Moreover, ML models can accurately predict sugar yields from SCB after different pretreatments, rapidly identifying high-performing conditions without exhaustive experimental screening (Al Azad et al., 2025). Within a circular bioeconomy framework, the convergence of conventional bioprocess engineering and AI creates multi-level synergy. Traditional methods supply physicochemical and biological fundamentals (pretreatment kinetics, enzyme kinetics, microbial metabolism), while AI tools contribute data-driven optimization, fault detection, predictive maintenance, and decision support across the value chain (Ubando et al., 2020; Marquez et al., 2026). Thus, AI and conventional approaches play complementary roles: AI delivers real-time control, predictive analytics, and optimization; traditional processes provide core conversion mechanisms that transform SCB into biofuels, biochemicals, and bio-based materials (Al Azad et al., 2025; Mehdizadeh et al., 2025).

Given these developments, integrating AI with conventional methods in SCB biorefineries is timely and essential. Experimental and data-driven methodologies together identify gaps in scale-up, techno-economic analysis, and life-cycle assessment, while outlining research directions to maximize SCB valorization within a circular bioeconomy. This paves the way for systematically combining conventional unit operations with AI-based optimization tools, transforming SCB from a low-value residue into a cornerstone of sustainable, circular, bio-based industrial systems (Kamboj et al., 2024; Garg et al., 2025).

**FIGURE 1:**
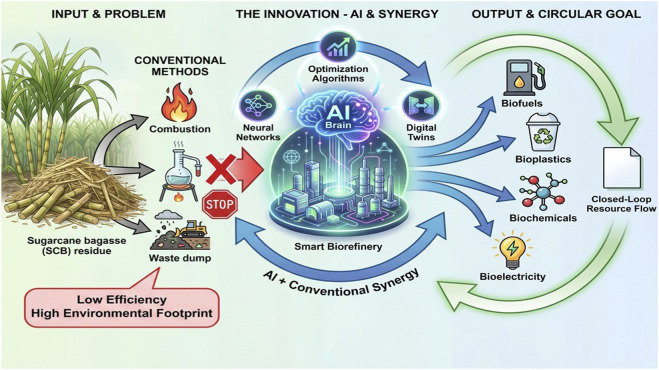
A conceptual framework for transitioning sugarcane bagasse (SCB) valorization from conventional methods to an AI-driven circular bioeconomy. Created with platform Sci-draw.com; then annotated and manually formatted using Microsoft PowerPoint.

Several previous reviews have addressed either SCB valorization without an AI focus (Kamboj et al., 2024; Silva et al., 2025) or AI applications in lignocellulosic biomass without focusing on SCB (Garg et al., 2025). Others have discussed circular bioeconomy concepts without a detailed comparison of conventional versus AI-driven methods (Teferi et al., 2025). To fill this gap, the present review offers a unified, systematic comparison between conventional bioprocessing strategies and AI-driven tools specifically for SCB valorization. It proposes an AI-centered circular bioeconomy framework where data-driven methods enable higher resource efficiency, reduced waste, and integrated value-stream utilization. The review also provides a critical gap analysis linking conventional biorefining limitations to targeted AI-based solutions, thereby bridging AI, biorefining, and sustainability. The overall conceptual framework is illustrated in Figure 1. Finally, this review aims to critically compare and integrate AI with conventional methods in SCB biorefineries, demonstrating how AI enhances process modeling, control, and optimization across major unit operations while highlighting synergies with traditional bioprocess engineering. It also identifies key challenges and future directions needed to establish SCB biorefineries as sustainable, scalable platforms, examines various pretreatment methods, and discusses opportunities for further research and practical application.

## 2 Environmental concerns of SCB

The current energy and environmental crises urgently demand sustainable alternatives to fossil fuels (Ramos et al., 2022). The sugarcane industry generates huge amounts of SCB worldwide (Chaudhary et al., 2021). A large fraction of bagasse is openly burned, endangering ecosystems and violating environmental regulations (Balaji et al., 2014). Bagasse has a calorific value of 1920 kcal kg^−1^ and is used in boilers for power generation. Yet untreated fly ash may contain toxic metals, causing secondary air pollution and harming humans, plants, and animals (Chandel et al., 2012). To achieve a cleaner industry, a shift toward environmentally friendly systems is necessary. SCB represents a sustainable biomass that can significantly reduce pollution and serves as an ideal feedstock for producing value-added products with advanced technological applications, for example, biochar (Ramos et al., 2022).

## 3 Sugarcane bagasse biorefinery

**FIGURE 2:**
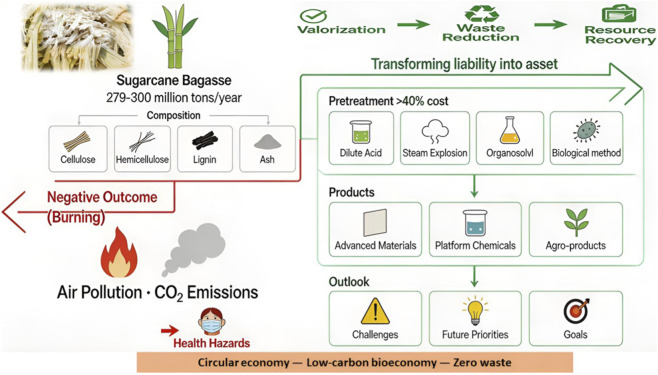
Illustrates the sustainable valorization of SCB: composition, pretreatment, environmental impact, bioproducts, and future perspectives. The figure highlights key challenges, research priorities, and sustainability goals aligned with circular economy, low-carbon bioeconomy, and zero-waste principles. Created with BioRender.com, then annotated and manually formatted using Microsoft PowerPoint.

The main characteristics of SCB global production (>100 Mt/year), chemical composition (cellulose, hemicellulose, lignin, and ash), environmental problems from open burning, and potential product streams are summarized in Figure 2. Traditional disposal by burning causes air pollution, CO_2_ emissions, and health hazards, while pretreatment alone accounts for over 40% of processing costs. Despite these challenges, modern sugarcane biorefineries have moved beyond just sugar and ethanol toward a circular bioeconomy model, already co-producing electricity and sometimes biogas (Formann et al., 2020). Thanks to its large-scale availability, SCB is a key feedstock for diverse biorefinery concepts (Silva et al., 2025). Advanced technologies now allow recovery of purified cellulose, nanofibers, and extracellular polymers, expanding SCB use into bioplastics, pharmaceuticals, food packaging, and water treatment (Teferi et al., 2025). Biotechnological valorization further supports production of biofuels, enzymes, and high-value products for a low-carbon circular economy (Silva et al., 2025).

Beyond individual products, bagasse can drive systemic change. Better CO_2_ utilization or sequestration through a circular sugarcane economy could de-fossilize other industries by supplying renewable biomaterials from complete residue exploitation (Formann et al., 2020). Economically, SCB valorization boosts industrial efficiency, creates rural jobs, and advances circular bioeconomy goals (Teferi et al., 2025). Nevertheless, major hurdles remain: high pretreatment costs, biomass recalcitrance, and the lack of industrial-scale conversion technologies (da Silva Aires et al., 2025). Integrated biorefineries are still challenging to implement, and many downstream processes require further improvement (Formann et al., 2020).

## 4 Traditional methods in SCB biorefining

Conventional lignocellulosic biorefining has long supplied the core pretreatment, hydrolysis, and fermentation steps for first-generation concepts (Agbor et al., 2011). Early bagasse processing favored low-selectivity, robust operations over high-value product differentiation (Agbor et al., 2011). Pretreatment methods: physical, chemical, physicochemical, and biological overcome biomass recalcitrance but differ in sugar yield, energy demand, and inhibitor formation; the choice of conditions affects economics and calls for integrated strategies in multi-output biorefineries (Agbor et al., 2011).

Conventional modeling, such as predicting glucose yield from SCB under varying enzyme loadings, enabled early process optimization (Gama et al., 2017). Such mechanistic models depend on a thorough biochemical understanding (Pistikopoulos et al., 2021). Superstructure optimization that combines techno-economic assessment with life-cycle analysis has been applied to organic waste valorization (Durkin et al., 2024). Traditional biological routes (fermentation, enzymatic hydrolysis) remain reliable and scalable for waste utilization within a circular bioeconomy (Hor et al., 2022). Major barriers to commercial lignocellulosic biofuels include high costs, complex supply chains, and recalcitrance, making them more expensive than fossil fuels. Therefore, improvements in energy efficiency, enzyme performance, and inhibitor tolerance are urgently needed (Balan, 2014). Continued innovation and scale-up, supported by co-product integration and favorable policies, are essential to lower costs and accelerate market entry.

### 4.1 Traditional pretreatment methods

#### 4.1.1 Physical pretreatments

Use mechanical forces (milling, electromagnetic waves, ultra-sonication, *etc.*) to break down biomass, increase surface area, and reduce crystallinity. Despite their effectiveness, they often suffer from low efficiency and high-energy consumption (Supplementary Table S1).

#### 4.1.2 Chemical pretreatments

Dissolve lignin and hemicellulose, thereby improving saccharification efficiency. Approaches include dilute acid, organic solvents, alkaline solutions, *etc.* Acid hydrolysis breaks hemicellulose into sugar monomers, while alkaline pretreatment and biodelignification remove lignin, leaving cellulose and hemicellulose accessible (Supplementary Table S1).

#### 4.1.3 Biological processes

Use enzymes or microorganisms to degrade lignocellulose (Supplementary Table S1). They consume less energy and produce fewer inhibitors than chemical or physical methods, but the long degradation time hinders industrial adoption. Enzymatic scarification can be performed as simultaneous saccharification and fermentation (SSF) or separate hydrolysis and fermentation (SHF), with SHF allowing separate optimization of pH and temperature for yeast and enzymes. A detailed summary of the advantages and disadvantages of physical, chemical, physicochemical, and biological pretreatments commonly applied to SCB is provided in Supplementary Table S1.

### 4.2 Mechanistic understanding, its limitations, and the role of AI

Conventional pretreatment and hydrolysis models rely on mechanistic principles, mass transfer, reaction kinetics (e.g., Michaelis-Menten), heat transfer, and thermodynamic equilibria, expressed as differential equations with physically meaningful parameters. When well understood, these models provide transparent, interpretable, and extrapolatable predictions, making them valuable for the design and scale-up of well-characterized systems (Agbor et al., 2011; Balan, 2014). However, such models struggle with complex lignocellulosic systems like SCB for several reasons. First, interactions among temperature, pH, enzyme loading, and substrate properties are highly nonlinear and difficult to capture with traditional kinetics. Second, many key parameters are unknown or vary with feedstock source, pretreatment history, and batch variations, while experimental measurement is costly and time-consuming. Third, mechanistic models assume homogeneity, ignoring real-world heterogeneity such as non-uniform particle size, local pH gradients, and enzyme deactivation. As a result, empirical approaches like response surface methodology (RSM) remain widely used, despite being labor-intensive and often missing the global optimum (Pistikopoulos et al., 2021). In contrast, AI and ML offer genuine advantages. Data-driven models (ANN, random forest, *etc.*) learn patterns directly from experimental data without requiring explicit equations, excelling at capturing nonlinear interactions, handling high-dimensional inputs, and adapting to new data. Specifically, AI can model complex processes when mechanistic understanding is incomplete, predict optimal conditions beyond initial experiments, serve as fast surrogate models for computationally expensive simulations, and enable real-time adaptive control using sensor data (Fischer et al., 2017; Al Azad et al., 2025). The synergy between conventional and AI approaches lies in combining their strengths rather than replacing one with the other. Mechanistic models provide a physically consistent foundation, generate synthetic training data, and guide feature selection, while AI learns residual nonlinearities, optimizes uncertain parameters, or acts as a correction term. Hybrid (mechanistic + data-driven) models are increasingly recognized as a powerful framework for SCB biorefineries, offering both interpretability and adaptability (Mountraki et al., 2020; Nair and Verma, 2025).

## 5 SCB as a platform for high-value bioproducts

**FIGURE 3:**
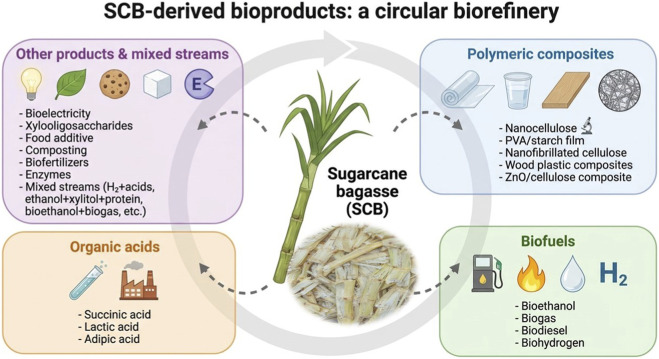
Comprehensive overview of SCB-derived bioproducts based on Supplementary Table S2. The central icon represents sugarcane bagasse (SCB), surrounded by four main product categories: polymeric composites (blue), second-generation biofuels (green), organic acids (orange), and other high-value products and mixed streams (purple). This circular layout illustrates the biorefinery concept and the diversity of value-added products obtainable from SCB. Created with BioRender.com, then annotated and manually formatted using Microsoft PowerP.oint.

SCB is a low-cost renewable feedstock that yields diverse high-value products: nanocellulose composites, bioadsorbents, biofuels, organic acids, food additives, enzymes, and biofertilizers. Steam explosion or alkaline pretreatment fractionates SCB into cellulose, hemicellulose, and lignin, producing nanofibrillated (NFC) and nanocrystalline (CNC) cellulose for biodegradable antimicrobial films and composites in packaging, textiles, and biomedical applications. Moreover, SCB-based green composites, such as ZnO–cellulose fabrics and wood-plastic hybrids, offer low density, thermal insulation, and a viable plastic replacement. Delignified residues generate bioadsorbents for dye and metal removal, whereas SCB–MoS_2_ hybrids exhibit high adsorption, photocatalysis, and antibacterial activity. For energy applications, SCB enables second-generation bioethanol, biogas, biodiesel precursors, and biohydrogen, often integrated with microbial fuel cells. In addition, organic acids (succinic, lactic, and adipic) derived from SCB serve as biodegradable polymer monomers. Turning to food and health, researchers have demonstrated the production of prebiotic xylooligosaccharides, phenolics, dietary fiber, β-carotene, and enzymes *via* solid-state fermentation. Finally, SCB composts into biofertilizers or acts as a carrier for phosphate-solubilizing microbes, directly linking agricultural waste to circular economy goals. A comprehensive list of these bioproducts, including polymeric composites, second-generation biofuels, organic acids, bioelectricity, xylooligosaccharides, food additives, enzymes, compost, biofertilizers, and nanocellulose composites, together with their pretreatment techniques, characterization methods, and references, is provided in the Supplementary Material (Supplementary Table S2; Figure 3).

### 5.1 SCB-based cellulose, nanomaterials, and sustainable packaging

Beyond its well-established role in biofuels and biochemicals, SCB is increasingly recognized as a sustainable source of cellulose for regenerated fibers, high-performance composites, and circular textile applications. Regenerated cellulosic fibers, such as viscose and lyocell, can be produced from SCB-derived cellulose pulp. Unlike the conventional viscose process, which relies on hazardous carbon disulfide (CS_2_), the lyocell process uses the recyclable solvent N-methylmorpholine N-oxide, thereby enabling closed-loop solvent recovery (Plakantonaki et al., 2022). Consequently, high-purity cellulose extracted from SCB meets fiber-spinning requirements, offering a sustainable alternative to wood pulp and cotton; these fibers are breathable, biodegradable, and suitable for clothing, home textiles, and non-woven products. In addition to regenerated fibres, SCB serves as a versatile platform for nanocellulose. Through controlled hydrolysis, mechanical fibrillation, or enzymatic treatments, cellulose nanocrystals and cellulose nanofibres can be isolated. These nanomaterials exhibit high specific surface area, low density, excellent mechanical strength, and good thermal stability. When incorporated into polymer matrices such as polyvinyl alcohol (PVA), polyhydroxybutyrate (PHB), or polylactic acid (PLA), SCB-derived nanocellulose significantly enhances mechanical and barrier properties, making the resulting composites suitable for biodegradable packaging, disposable tableware, and even automotive components (Yang et al., 2020; Ali et al., 2022). The fashion industry, which seeks sustainable alternatives to petroleum-based synthetics and water-intensive cotton, also benefits from SCB-based regenerated fibers. These fibers require less water and land than cotton cultivation, and their production valorizes an agricultural residue that would otherwise be burned or landfilled, thereby reducing pollution and greenhouse gas emissions. Thus, this approach aligns directly with circular textile economy principles, where waste from one industry becomes a resource for another.

Another rapidly growing application is biodegradable packaging. Films, coatings, and trays have been developed from native SCB cellulose or nanocellulose. Importantly, these materials can be engineered with active or intelligent functions, antimicrobial activity (*via* ZnO nanoparticles, nisin, or essential oils) or oxygen scavenging, to extend food shelf life (Chen et al., 2020; Ali et al., 2022). Unlike conventional plastics that persist for centuries, SCB-based packaging is compostable and degrades within weeks to months, making it an attractive alternative for single-use containers, shopping bags, and agricultural mulch films. Taken together, regenerated cellulose fibers, nanocellulose composites, textile materials, and biodegradable packaging from SCB reduce reliance on fossil-based materials, valorize agricultural waste, create biodegradable products, and add value to sugarcane residues. A comprehensive list of these bioproducts, along with corresponding references, is provided in Supplementary Table S2. As production technologies mature, SCB is expected to become a key feedstock for the global bio-based materials industry.

## 6 Role of AI in biorefinery processes

AI is transforming SCB biorefinery operations through data-driven optimization. For instance, Fischer et al. (2017) showed that ML models, artificial neural networks (ANN), C5.0 classification trees, and random forests predict ethanol production with high accuracy (*R* ^2^ > 0.90, absolute deviation <8.1%), while optimizing temperature, enzyme loading, biomass, inoculum, and fermentation time. Recent trends have shifted from purely physical modeling toward data-driven approaches; AI mimics human cognition *via* heuristic algorithms, ML, and fuzzy logic (Olawuni et al., 2024), and it confirms nonlinear input-output relationships critical for biorefining (Luo et al., 2021).

In anaerobic digestion, ANNs predict methane production with RMSE as low as 0.001 and *R* ^2^ up to 0.99, and they can be trained without prior mechanistic understanding (Fard and Pouramini, 2022; Metwally et al., 2024). Beyond optimization, AI enables real-time monitoring, quality control, predictive maintenance, reduced energy and waste, and maximized feedstock use (Orejuela-Escobar et al., 2024). Moreover, AI-assisted catalyst design cuts greenhouse gas emissions by up to 40% (Damian et al., 2026). Combining AI with traditional methods also works well; for example, orthogonal design coupled with ML maximized cellulose/hemicellulose recovery while minimizing validation errors (Nair and Verma, 2025). Looking forward, synthetic biology, genomics, AI, and chemistry will enable AI-informed smart biorefineries (Yu et al., 2026). Additionally, surrogate models blending data-driven methods with conventional flowsheet models add further sophistication (Mountraki et al., 2020). The key AI techniques discussed above are summarized in Table 1, along with their reported improvements, limitations, and estimated TRL.

**TABLE 1:** Illustrative examples of AI/ML techniques applied to sugarcane bagasse (SCB) biorefinery processes, with reported improvements, key limitations, and estimated technology readiness level (TRL).

AI technique	Target process	Reported improvement	Key limitations	TRL (est.)	References
Artificial Neural Networks (ANN)	Delignification (alkaline H2O2)	Accurate prediction of lignin removal, glucose, and xylose concentrations (low RMSE, R2 close to 1).	Requires large datasets; black-box nature; risk of overfitting	4 (lab)	Rego et al. (2018)
Random Forest (RF)	Pretreatment + enzymatic hydrolysis	High prediction accuracy (*R* ^2^ > 0.90); reduces experimental waste by 45%	Less accurate for highly non-linear relationships; still requires moderate data	4 (lab)	Al Azad et al. (2025)
Genetic Algorithm (GA)	Multi-objective optimization (ethanol via gasification + syngas fermentation)	Pareto-optimal trade-offs between cost, energy, and emissions	Computationally expensive; no inherent predictive model	4 (simulation)	de Medeiros et al. (2021)
GA	Anaerobic digestion of sugar bagasse	Maximized biomass production; outperformed RSM	Limited to optimization; does not model process dynamics	3 (proof-of-concept)	Saeid et al. (2023)
Adaptive Neuro-Fuzzy Inference System (ANFIS)	Enzymatic hydrolysis (glucose yield)	*R* ^2^ = 0.9992 (higher than RSM’s 0.9859); best conditions: 60 h, 3.3% enzyme, 23.3 g/L substrate	Complex design; still requires sufficient data	3 (proof-of-concept)	Boico et al. (2025)
Hybrid ANN-GA	Dark fermentation (bio-H2)	Minimal prediction error (0.02); optimal parameters: 48.98 g/L substrate, 8.21% inoculum, 3.56% acid, pH 7.02	High computational cost; careful parameter tuning required	4 (lab)	Raut et al. (2025)
Rule-based ML (ensemble)	Pretreatment + enzymatic hydrolysis	Glucose yield 84.3%, xylose yield 63.3%, delignification 92.3%; waste reduction 45%	Limited to training data range; industrial validation pending	4 (lab)	Al Azad et al. (2025)

TRL estimates are based on the authors’ assessment of the reported studies (laboratory-scale validation = 3–4; pilot-scale = 5–6; industrial = 7–9). Most AI, applications in SCB literature remain at TRL 3–4.

While the results summarized in Table 1 demonstrate the clear potential of AI/ML for SCB biorefinery optimization, several critical limitations must be acknowledged. Most reported high *R* ^2^ values are obtained on relatively small, laboratory-generated datasets; performance often drops when models are applied to independent or industrial data, indicating overfitting or lack of generalizability. Many studies do not provide open access to their code or data, making reproducibility difficult. The “black-box” nature of deep neural networks limits interpretability, which is a major obstacle for industrial adoption where operators require explainable decisions. The scarcity of large, high-quality, publicly available datasets for SCB processes remains a fundamental bottleneck. Consequently, hybrid approaches that combine mechanistic knowledge with data-driven ML are increasingly advocated, as they offer better extrapolation capability and require less training data. However, bridging the “lab-to-fab” gap remains a major hurdle, as the majority of AI-optimized conditions have not been experimentally validated beyond the original study, and the technology readiness levels (TRL) of most proposed models remain at the laboratory scale (TRL 3–4) due to persistent challenges in data standardization and model transparency (Butean et al., 2025; Garg et al., 2025). Closing this gap will therefore require a concerted effort that includes standardized benchmarking, the creation of open datasets, and rigorous cross-validation on diverse SCB varieties and pretreatment histories.

Nevertheless, response surface methodology (RSM) and other classical design-of-experiment methods have long served as effective tools for process optimization in lignocellulosic biorefineries, particularly when experimental budgets are limited or the number of variables is small (Agbor et al., 2011; Balan, 2014). RSM typically requires far fewer experiments than conventional ML approaches, does not rely on large datasets or specialized programming skills, and often provides sufficient predictive accuracy for practical purposes (Pradhan et al., 2022; Nair and Verma, 2025). In such resource-constrained contexts, the added complexity and data demands of AI/ML may not be justified. Consequently, claims that AI “outperforms” classical methods should be interpreted cautiously; the performance gap depends strongly on the specific problem, the quality of RSM implementation (design, replication, validation), and data availability. A fair comparison requires both ML and RSM models to be optimized to a similar degree, a condition that is not always met in the published literature (Pradhan et al., 2022; Nair and Verma, 2025).

### 6.1 Optimization of pretreatment and hydrolysis

Pretreatment and hydrolysis are critical bottlenecks in lignocellulosic biorefineries, yet conventional trial-and-error optimization fails to capture the complex nonlinear interactions among temperature, reagent concentration, time, solid-to-liquid ratio, and enzyme loading. Consequently, researchers have increasingly turned to AI and ML methods, ANNs, support vector machines, random forests, and Bayesian optimization, which accurately model and optimize these steps with far fewer experiments (Al Azad et al., 2025; Nair and Verma, 2025). For example, Valim et al. (2017) used ANN to model alkaline H_2_O_2_ delignification of SCB (25 °C–45 °C, 1.5%–7.5% H_2_O_2_). The ANN predicted lignin oxidation with high accuracy, identifying 25 °C and 4.5% H_2_O_2_ as the optimum, which experimentally removed ≈54% of lignin. Similarly, Al Azad et al. (2025) employed Random Forest, XGBoost, and ANN to optimize dilute acid/alkaline pretreatment (120 °C–180 °C, 30–120 min) followed by enzymatic hydrolysis. Their ML-optimized process achieved >80% glucose yield (75 g/L) while cutting experimental runs by 45% compared to RSM, with projected economic gains of $321 million by 2025 and $494 million by 2030.

A different approach came from Gitifar et al. (2013), who compared dilute acid alone versus dilute acid plus ozonolysis; adding ozonolysis boosted glucose yield about fourfold, and ANN with k-fold cross-validation accurately predicted glucose concentration. Beyond glucose, Ogedjo et al. (2022) compared RSM, ANN, and a fuzzy inference system for levulinic acid production; both ANN and the fuzzy system outperformed classical RSM in prediction accuracy. Likewise, Boico et al. (2025) compared RSM with an adaptive neuro-fuzzy inference system (ANFIS) for enzymatic hydrolysis, finding that ANFIS provided more accurate predictions and better captured nonlinear dynamics.

Extending to materials science, Khan et al. (2025) developed a strain-rate-dependent optimization framework for alkali treatment of SCB fibers using ensemble learning and a genetic algorithm, reducing trial-and-error and chemical use. In process control, Furlong (2015) designed an automated reactor with an ANN-based soft sensor for real-time glucose inference, embedded in a closed-loop control system. Complementing this, Moreira Neto et al. (2023) applied an ANN-inspired genetic algorithm (Pikaia) to estimate kinetic parameters of a semi-mechanistic hydrolysis model, creating a data-driven, AI-assisted simulation framework. Finally, Fischer et al. (2017) used ANN, a C5.0 classification tree, and random forest to model ethanol production in simultaneous hydrolysis and fermentation (SHF). These models achieved *R* ^2^ > 0.90, identifying near-optimal conditions (≈35 °C, 36 h, 99.8% enzyme load, 29.5 g/L inoculum, 24.9% bagasse) that yielded 12.1 g/L ethanol with 0.336 g/L·h productivity. Taken together, these studies demonstrate that ML techniques are powerful, data-driven tools for understanding, predicting, and optimizing lignocellulosic ethanol biorefineries. A summary of key quantitative improvements is presented in Table 2.

**TABLE 2:** Quantitative performance improvements achieved by AI/ML in SCB biorefinery processes.

Biofuels and platform chemicals production			
Performance indicator	Reported improvement/Outcome	AI/ML technique used	References
Glucose yield	>80% (75 g/L) from pretreated SCB	Random Forest, XGBoost, ANN	Al Azad et al. (2025)
Glucose yield (modeling)	*R* ^2^ = 0.9992 (ANFIS) vs. 0.9859 (RSM)	ANFIS, RSM	Boico et al. (2025)
Xylose yield	63.3% from pretreated SCB.	Random Forest, XGBoost, ANN	Al Azad et al. (2025)
Ethanol concentration	12.1 g/L; optimized at 35 °C, 36 h, 24.9% bagasse	ANN, Random Forest, C5.0	Fischer et al. (2017)
Ethanol productivity	0.336 g/L·h	ANN, Random Forest, C5.0	Fischer et al. (2017)
Industrial bioethanol	10% increase in concentration and production	ANN + Particle Swarm Optimization (PSO)	Pereira et al. (2021)
Levulinic acid	5.40 mg/mL yield (77.1% efficiency); ANN *R* ^2^ = 0.96	ANN, RSM, FIS	Ogedjo et al. (2022)
Biohydrogen (Bio-H_2_)	Prediction error of 0.02; optimal conditions identified	ANN + Genetic Algorithm (GA)	Raut et al. (2025)
Methane production	RMSE = 0.001, *R* ^2^ = 0.99	ANN	Metwally et al. (2024)
GHG Emission Reduction	Up to 40% reduction	AI-assisted catalyst design	Damian et al. (2026)

Process efficiency and feature importance			
Reduction in experimental runs	45% reduction compared to traditional RSM	Random Forest, XGBoost, ANN	Al Azad et al. (2025)
Delignification (Lignin removal)	*R* ^2^ near 1 for predictive accuracy	ANN	Valim et al. (2017)
Pretreatment	Cellulose recovery: time (26.2%) Hemicellulose recovery: temp (37.5%) Delignification: solid loading (19.6%)	SHAP analysis on ML model	Al Azad et al. (2025)
Hydrolysis	Glucose yield: substrate loading (45.7%) Xylose yield: substrate loading (37.8%)	SHAP analysis on ML model	Al Azad et al. (2025)

### 6.2 AI for smarter process control

AI transforms biorefinery feedstock management into a dynamic, circular-driven system. ML models digest real-time data (composition, moisture, seasonality, contaminants) and assign optimal valorization routes, boosting resource efficiency while cutting waste and emissions (Orejuela-Escobar et al., 2024). Moreover, AI-driven control frameworks merge soft sensors, predictors, and multi-objective solvers to auto tune operations, spot bottlenecks, and switch between conversion pathways, enabling operators to stabilize units, balance productivity against environmental load, and embed sustainability into control logic.

Clauser et al. (2022) present a critical review of how Industry 4.0 technologies—particularly AI, big data analytics, and the Internet of Things—can be integrated into biorefinery process design to advance the bioeconomy and circular economy agendas. The authors argue that modern manufacturing is shifting from traditional, static designs to dynamic, data driven systems, and that biorefineries must adopt these tools early in the design phase to achieve sustainable economic development. The review highlights that effective biorefinery design requires simultaneous optimization of technical, economic, environmental, and social factors, and that Industry 4.0 tools can support this by improving decision making, process selection, and biomass valorization strategies. The authors propose a novel methodology for sustainable process design that combines multi feedstock biorefinery synthesis with simultaneous optimization of economic and environmental targets, wrapped in a sustainability weighted return on investment metric (SWROIM) to evaluate tradeoffs. Overall, they frame Industry 4.0 as a key enabler for integrating advanced digitalization into biorefinery platforms, thereby enhancing resource efficiency, circularity, and the long term viability of bio based value chains.

Nasef et al. (2026) reported that neural networks and surrogate models learn from live sensor streams (feedstock, reactor state, and product flows), then reset set points and strategies on the fly, even under biomass or demand shifts, while enabling predictive maintenance and closed-loop tuning of pretreatment, hydrolysis, fermentation, and energy recovery.

Turning to anaerobic digestion, Metwally et al. (2024) proved that ANN beat classical models at predicting methane from bokashi-treated SCB. Embedded in an online system, the ANN continuously adjusts loading, retention, and mixing to stabilize the reactor and maximize energy capture. Likewise, Ghatak and Ghatak (2018) trained an ANN on sugarcane waste plus cattle dung; it forecasts biogas curves with high *R* ^2^, helping operators schedule feeding and avoid upsets. Furthermore, Ranade et al. (2021) applied ANN surrogates to hydrodynamic cavitation pretreatment of SCB, estimating hard-to-measure variables (cavitation intensity, radical yield) and allowing real-time adjustment of flow and pressure.

At the mill gate, Dos Santos et al. (2021) built an ANN-based image classifier that separates sugarcane stalks from soil, stones, and debris with near-perfect accuracy (100% for 90–100 wt% and 41–87 wt% sugar content). Low-cost and fast, it feeds real-time quality loops, enabling dynamic acceptance criteria, cleaning schedules, and crusher optimization, thereby boosting sugar extraction and process stability. Owusu and Marfo (2023) reviewed how ANNs, support vector machines, and ensemble models capture nonlinearity in pretreatment, hydrolysis, and fermentation, accurately predicting bioethanol yields. Coupled with genetic or particle swarm algorithms, they find near-optimal conditions with fewer trials. Consequently, these AI tools enable real-time monitoring, fault detection, adaptive control, and integration with techno-economic and life-cycle analysis, making AI indispensable for smart bioethanol refineries. Finally, de Jesus et al. (2024) used an ANN-GA hybrid to model dye removal by biosilica from SCB ash. Their hybrid outperformed classical empirical models in grasping nonlinear effects of pH, dose, and contact time, displaying SCB ash as a low-cost, eco-friendly adsorbent for wastewater treatment.

### 6.3 Integrating biorefinery design

#### 6.3.1 System optimization and control

AI, especially ANNs and adaptive neuro-fuzzy inference systems, drives integrated SCB biorefinery design. These tools capture nonlinear relationships between operating variables (pretreatment type, temperature, reagent dose, enzyme load, and fermentation time) and key outcomes (sugar yield, ethanol, biogas, organic acids, lignin, and thermal properties). They outperform classical regression and RSM in predictive accuracy. When combined with evolutionary algorithms like GA, particle swarm optimization, and ant colony optimization, they identify near-optimal windows that maximize output and efficiency (Pradhan et al., 2022). Garcia and Ensinas, (2024) developed a mixed integer linear programming superstructure that selects conversion technologies, scales them, and integrates heat recovery and utilities for SCB. Using AI-inspired mathematical programming, the model identifies heat-exchange zones, steam levels, and utility types that minimize annualized cost and energy demand while boosting heat synergy. Carpio et al. (2021) employed multi-objective particle swarm optimization to design first- and second-generation (1G/2G) ethanol biorefineries, simultaneously maximizing net present value and minimizing global warming potential with carbon-pricing integration, generating Pareto-optimal solutions. Emori et al. (2023) designed a neural network predictive controller for a four-stage evaporator in a 1G/2G plant, continuously adjusting steam flow to maintain sugar concentration despite disturbances. Although Zininga et al. (2024) presented a process-based platform (glycerol pretreatment, enzymatic hydrolysis, and fungal culture for lipids and polymers) rather than an explicit AI system, their rich dataset on pretreatment severity, sugar recovery, and product profiles is ideal for training ML models to predict optimal windows and balance competing outputs.

#### 6.3.2 Fuel, materials, and bioH_2_

In fuel purification, Olawuni et al. (2024) showed that AI optimizes adsorptive desulphurization using cellulose nanocrystals (CNC) as green adsorbents. AI models map temperature, pressure, contact time, and adsorbent dosage to sulfur removal efficiency while assisting CNC isolation from waste, enabling real-time adaptive control. For bio-methanol, Yousef et al. (2020) combined fuzzy logic with particle-swarm optimization to boost yield from SCB pyrolysis. Their fuzzy model fit experimental data better than a comparable ANN, and PSO increased predicted bio-methanol yield by about 20% above the best experimental runs, without hardware changes. In construction materials, Utami et al. (2025) used AI (ANN, RSM, GA) to optimize SCB ash as a cement substitute, balancing strength, workability, durability, and waste reduction; AI-guided design outperforms trial and error, promoting agro-waste recycling. Similarly, Singh and Patel (2023) combined experiments with ML (ANN, regression) to test SCB ash as a fine aggregate replacement, capturing nonlinear effects and identifying optimal substitution levels. Finally, Raut et al. (2025) built an AI framework for biohydrogen (bioH_2_) production from SCB using a novel *Alcaligenes ammonioxydans* strain. They integrated ANN, GA, and RSM in Python: ANN to model nonlinear relationships (substrate, pH, temperature, inoculum vs. H_2_ yield), GA to find global optima, and RSM for validation. Optimal conditions: substrate 48.98 g/L, inoculum 8.21% v/v, acid 3.56% v/v, and pH 7.02. This hybrid approach displays advanced optimization for sustainable bioH_2_ production.

## 7 Integration with circular bioeconomy

AI combined with conventional SCB biorefinery methods drives the circular bioeconomy by turning waste into resources. For instance, the biological use of cellulosic ethanol waste streams is already circular (Hor et al., 2022). AI enables predictive modeling, catalyst design, real-time monitoring, and predictive maintenance, thereby improving efficiency and enabling high-value bioproducts (Orejuela-Escobar et al., 2024). Damian et al. (2026) showed that AI-assisted green catalysts achieved over 90% conversion, 98% dye degradation, and 40% GHG reduction.

Beyond these achievements, AI and ML are increasingly applied to catalyst design for lignocellulose conversion, directly supporting GHG emission reductions. For example, Madadi et al. (2025) developed an ML-driven framework using 3,451 data points from 54 studies to model lignin monomer production, achieving R = 0.80–0.86 and projecting a CO_2_ reduction of 20.6 million tons per year. Similarly, Sun et al. (2025) designed a closed-loop ML workflow for biomass-derived porous carbons, producing a SCB-derived material (Al-S) with a CO_2_ working capacity of 12.3 mmol/g at 273 K and 10 bar, the highest reported. Moreover, Zhang et al. (2025) employed automated machine learning (AutoML) to model biomass catalytic pyrolysis, achieving *R* ^2^ > 0.912 and using reverse optimization to identify Zn-modified zeolite as the optimal catalyst with ideal conditions (550 °C–650 °C, 1–3 wt% metal, Si/Al ratio 30–40, H/C ratio 1.4–1.6). These studies collectively illustrate that AI-guided catalyst design and process optimization deliver measurable environmental benefits.

Beyond catalyst design, AI also optimizes batch sizes and product mixes (Estarriaga-Navarro et al., 2026) and improves biowaste processing (Nair and Verma, 2025). SCB valorization strengthens industry and circular goals, with advanced extraction of cellulose, nanofibers, and polymers for bioplastics, pharmaceuticals, and packaging (Teferi et al., 2025). Predictive maintenance reduces unplanned failures and off-spec batches, cutting material waste, while real-time adaptive control adjusts to feedstock variability, maximizing yield and minimizing residuals. However, efficiency gains do not automatically equal circularity; true circularity requires reintegrating waste streams (e.g., water recycling, lignin conversion). Most AI studies report efficiency gains, not direct circularity metrics. A rigorous assessment needs indicators such as material circularity rate (MCR), end-of-life recovery fraction, or virgin resource reduction. Although Table 3 outlines key circularity indicators and the potential improvements achievable through AI-driven control, no AI-optimized SCB process has to date been evaluated against these metrics.

**TABLE 3:** Key circularity indicators and how AI-driven control can improve them in SCB biorefineries.

Circularity indicator	What it measures	How AI can improve it in SCB biorefineries	References
Material circularity rate (MCR)	Share of non-virgin material in the total material input (including recycling)	Optimize blend ratios of SCB-derived intermediates and co-products, minimizing virgin feedstock demand.	Shah et al. (2025)
End-of-life recovery fraction	Fraction of SCB-derived products recovered at end of use (e.g., bioplastics, biopolymers)	Predict degradation pathways and optimize product design for reuse/recycling.	Shah et al. (2025)
Product recovery ratio (biorefinery side)	Mass of recovered high-value products (ethanol, xylitol, enzymes) vs. input SCB	Use ML-based yield prediction and closed-loop control to minimize off-spec batches and maximize product streams.	Elgarahy et al. (2025)
Water and nutrient recycling rate	Fraction of water and process nutrients that are reclaimed and reused	Predict fouling and scaling, trigger timely cleaning, and dynamically adjust recycling loops.	Liu et al. (2025)
Waste-to-energy closure rate	Fraction of SCB residues converted to energy or intermediates instead of disposal	Optimize cogeneration and anaerobic digestion parameters to maximize energy recovery from residues	Metwally et al. (2024), Elgarahy et al. (2025)

References are representative; the reader is referred to the main reference list for full citations.

As shown in Table 3, AI-guided control can increase material circularity, enhance end-of-life recovery, improve product recovery, sustain water-nutrient recycling, and maximize waste-to-energy closure. Nevertheless, most published studies do not report such metrics or quantify virgin material savings (Orejuela-Escobar et al., 2024; Nair and Verma, 2025). Full SCB valorization should integrate food, health, and industrial applications (Teferi et al., 2025), while AI can align bio-based production with the Sustainable Development Goals (Raman et al., 2025). Only when AI-driven gains are tied to well-defined circularity indicators can the transition to regenerative, low-carbon SCB biorefineries be meaningfully assessed.

Consequently, the link between AI and circularity remains underexplored. Future studies should adopt a standardized protocol: (i) report MCR; (ii) specify end-of-life recovery and product recovery ratios; (iii) report water/nutrient recycling rates; (iv) compare AI results against a non-AI baseline; (v) annotate datasets with circularity tags. Moreover, we encourage coupling AI optimization with life-cycle assessment. Finally, green chemistry, material innovation, and data-driven engineering will define next-generation biorefineries, requiring a multidisciplinary blend of biotechnology, materials science, and AI (Nair and Verma, 2025).

### 7.1 Limitations of AI models for SCB biorefineries

Although AI and ML hold promise for improving yields and process optimization in SCB biorefineries, several limitations constrain their practical impact. First, ML models require large, high-quality, well-annotated datasets, yet such datasets remain scarce and heterogeneous in SCB research due to variations in feedstock composition, pretreatment protocols, and reporting standards (Mountraki et al., 2020; Orejuela-Escobar et al., 2024; Nair and Verma, 2025). Small sample sizes and inconsistent metadata amplify noise and bias, limiting model generalizability.

Second, overfitting is a recurrent risk: high reported metrics (e.g., *R* ^2^) often reflect fitting to training data idiosyncrasies rather than robust predictive power. Without rigorous validation, k-fold cross-validation, independent test sets, and transparent reporting, performance can be misleading (Fischer et al., 2017; Fard and Pouramini, 2022). Third, ML models interpolate well within the training domain but extrapolate poorly; conditions outside the original data range (novel pretreatments, scale changes, feedstock variability) produce unreliable predictions. Hence, surrogate models must be used conservatively or combined with mechanistic knowledge (Mountraki et al., 2020; Nair and Verma, 2025).

Fourth, many reported “optimizations” remain purely computational; without wet-lab confirmation, algorithmic optima may be infeasible or unsafe when implemented (Metwally et al., 2024; Damian et al., 2026). Case studies document both successes and failures where *in silico* suggestions were not experimentally validated (Olawuni et al., 2024; Orejuela-Escobar et al., 2024; Yu et al., 2026).

Beyond these issues, data provenance and methodological transparency are critical. Most cited studies rely on proprietary or inaccessible datasets, preventing independent validation and replication (Nair and Verma, 2025). Moreover, details on data preprocessing (missing values, outlier removal, normalization, feature engineering) are frequently omitted, affecting model outcomes and cross-study comparability. Likewise, many studies do not clearly report train-test splitting or validation protocols; cross-validation is sometimes used without specifying folds or nesting. Few perform external validation on truly independent datasets. Consequently, reported metrics may reflect overfitting rather than genuine predictive power for unseen conditions. Finally, practical constraints, data privacy, inconsistent reporting standards, limited resources for large-scale validation, and lack of interdisciplinary expertise further slow translation from model to plant. To improve credibility, future work should adopt transparent reporting guidelines, including open data and code, clear preprocessing descriptions, explicit split definitions, and external validation where possible (Fischer et al., 2017; Al Azad et al., 2025). Thus, hybrid approaches that combine mechanistic knowledge with data-driven ML, together with rigorous validation protocols, are urgently needed to bridge the gap between laboratory promise and industrial practice.

### 7.2 Guidelines for selecting appropriate AI techniques for SCB processes

Different AI techniques offer distinct strengths; choosing the most suitable method depends on the problem, data availability, and desired outcome. Artificial neural networks (ANNs) excel at nonlinear prediction and optimization, learning complex relationships among pretreatment severity, enzyme loading, reaction time, and product yield. They are powerful when large, high-quality datasets are available and input-output relations are strongly nonlinear. However, ANNs are “black-box” models, require careful tuning to avoid overfitting, and demand substantial computational resources. They are generally preferred over Random Forest when datasets are large and interaction effects are highly nonlinear. Support vector machines (SVMs) are useful for relatively small datasets where a strong generalization boundary is needed. They capture nonlinear patterns *via* kernel functions but are less common in recent SCB literature due to scaling issues with large data. Random forests (RFs) are robust ensemble predictors that handle noisy data well and reveal variable importance. They are more effective for stable prediction than for finely resolving continuous response surfaces. RFs are a good choice when interpretability of variable importance is valuable and the relationship is moderately nonlinear; they are often preferred over ANNs when datasets are limited or when identifying influential inputs is the goal. Fuzzy inference systems (FIS), including ANFIS, are valuable when process knowledge is partly qualitative or uncertain, combining expert rules with data-driven learning. They offer better interpretability than pure ANNs and suit problems where both data and expert knowledge exist. Genetic algorithms (GAs) are optimization tools that search large parameter spaces to maximize yield or minimize cost. They complement neural networks because ANNs provide fast, accurate surrogates, allowing GAs to explore thousands of candidate conditions without costly experiments. This hybrid ANN-GA approach is especially effective for rugged, multi-modal optimisation landscapes. Digital twins integrate models with live process data to simulate, monitor, and control operations in real time (Fahmy et al., 2015; Varatharajah and Victor, 2016; You et al., 2017; Kumar et al., 2024; Asif et al., 2025). They require substantial infrastructure and are best suited for well-understood processes with reliable mechanistic models and continuous sensor data streams. A comparative summary of these techniques, with illustrative examples from SCB literature, is presented in Table 1.

## 8 Emerging industrial applications of AI in sugarcane processing

Although fully integrated, end-to-end AI-controlled SCB biorefineries remain at an early stage, a growing number of real-world implementations demonstrate measurable benefits in sugarcane processing facilities. Supplementary Table S3 summarizes documented industrial case studies illustrating this trend, based on industry reports, press releases, and conference proceedings. Nevertheless, an honest accounting of industrial deployment challenges is required. Despite the successes documented in Supplementary Table S3, most academic AI studies remain at laboratory scale (TRL 3–4), and very few have translated into practice. Real-time sensors frequently fail under harsh industrial conditions (high temperature, humidity, and fouling), leading to missing or unreliable data streams, a situation that laboratory models typically ignore (Brunner et al., 2021). Furthermore, process data often contain missing values, outliers, and inconsistent sampling rates, yet most ML models assume clean, complete datasets (Brunner et al., 2021; Rhyu et al., 2024). In addition, feedstock variability in industrial settings regularly exceeds training distributions, causing unpredictable predictions (Narani et al., 2017). Control actuators also have physical constraints (limited range, dead time, and nonlinear response) that are rarely incorporated into laboratory-scale optimization studies (Luppi et al., 2019). Finally, the lack of standardized data infrastructure, high implementation costs, and industry resistance to black-box models further slow adoption. Thus, while the industrial examples in Supplementary Table S3 demonstrate that AI can succeed at TRL 8–9 in specific unit operations, end-to-end AI-controlled SCB biorefineries remain a future aspiration. Bridging this gap requires collaborative efforts between academia and industry, open datasets, and validation protocols that explicitly account for real-world constraints.

## 9 Challenges, limitations, and future directions

Despite the potential of integrating AI with conventional SCB biorefinery methods, several obstacles remain. Technical and economic barriers persist, including high pretreatment costs, lignocellulosic recalcitrance, and the absence of industrial-scale conversion technologies (Silva et al., 2025). Moreover, bagasse’s complex structure demands expensive pretreatments, limiting competitiveness against fossil inputs. Regulatory and policy gaps–such as absent incentives and unclear regulations–further hinder market entry for SCB-derived products (Silva et al., 2025). Consequently, market penetration remains constrained even when technical feasibility is proven, affecting biorefinery economics and limiting agricultural waste’s contribution to resource circularity. Turning to economic realities, the $321 million projection by Al Azad et al. (2025) from ML-optimized SCB processes was based on laboratory data under ideal conditions, ignoring substantial capital and operational costs. Economic realities of AI adoption represent another critical challenge. Detailed cost-benefit analyses specific to SCB biorefineries are largely absent from the peer-reviewed literature, which in itself constitutes a significant research gap. Industry reports suggest that AI-native greenfield facilities require substantially higher upfront investment than retrofitting existing plants, and that retrofitting sensors is considerably more expensive than embedding them during original construction. Workforce upskilling is another major expense that is rarely accounted for in academic studies. Consequently, laboratory-derived projections of economic gains, such as the $321 million figure reported by Al Azad et al. (2025), lack the necessary real-world economic context. Documented industrial deployments (e.g., the MM.IA system in Brazil) have generated substantial additional revenue, but their full cost structures have not been publicly disclosed. These examples indicate that while AI can generate positive returns, the economic case is highly case-specific and requires detailed, location-dependent analysis. Another critical limitation concerns the fairness of comparisons between AI/ML and classical methods like response surface methodology (RSM). Many studies compare highly optimized ML models against default or poorly implemented RSM designs, biasing results in favor of ML. Where RSM is properly optimized (appropriate design, sufficient center points, and rigorous diagnostics), the performance gap narrows considerably; for low-dimensional, moderately nonlinear problems, RSM may even match or exceed ML accuracy. Hence, claims of AI superiority should be interpreted cautiously, and future studies should adopt standardized benchmarking protocols with equivalent optimization levels and transparent reporting.

Beyond technical and economic barriers, the circular bioeconomy framework carries implicit assumptions that may not benefit local communities. As Khanna et al. (2024) argue, the transition must balance circularity with its costs, benefits, and distributional effects. AI-controlled biorefineries redistribute risks (job displacement, environmental burdens) onto local populations while profits accrue to distant investors, necessitating transparent risk-sharing and community benefit agreements. Data ownership and algorithmic accountability remain unresolved: companies and black-box models obscure responsibility for harm typically own process data. Moreover, large-scale SCB valorization may divert biomass from traditional uses (animal feed, cooking fuel, fertilizer), undermining rural livelihoods (Wagh et al., 2024). A just transition requires social impact assessments, participatory policy design, and inclusion of local communities (Kacprzak et al., 2025). Only by addressing these social dimensions can SCB biorefineries contribute to a truly sustainable and equitable circular bioeconomy.

Looking forward, future advancement requires cost-effective, energy-efficient, and scalable green technologies (Teferi et al., 2025). Research should priorities low-energy pretreatments, advanced enzymatic hydrolysis, efficient solvent recovery, and expanded bagasse use in biodegradable packaging, animal feed, pollutant removal, and sustainable agriculture. The convergence of green chemistry, material innovation, and data-driven engineering will define next-generation biorefineries, requiring a multidisciplinary blend of biotechnology, materials science, and AI (Nair and Verma, 2025). To overcome economic and technical barriers, concerted investments in applied research, integrated biorefinery development, and academia-industry-government partnerships are needed (Formann et al., 2020; Silva et al., 2025). Crucially, future techno-economic analyses should integrate AI optimization with full life-cycle costing, including capital, infrastructure, training, and maintenance, to provide realistic guidance. By addressing these challenges, SCB can be transformed into a strategic, high-value feedstock driving innovation, resource circularity, and climate resilience within a sustainable bioeconomy (Teferi et al., 2025).

## 10 Conclusion

At the intersection of AI and sustainable agriculture, SCB has become a strategic asset for a low-carbon future rather than a waste burden. This review provides a unified framework demonstrating how AI, particularly ML, neural networks, and hybrid optimization algorithms, can overcome the inherent limitations of conventional SCB biorefineries. AI enables data-driven modeling, real-time adaptive control, predictive maintenance, and multi-objective optimization across pretreatment, hydrolysis, fermentation, and cogeneration. Importantly, AI does not replace conventional bioprocess engineering but complements it, yielding an intelligent and self-optimizing SCB biorefinery that closes material loops, diversifies products, and maximizes resource efficiency.

Nevertheless, several gaps hinder widespread adoption. Techno-economic barriers–high capital costs, insufficient regulatory support, and a lack of scalable demonstration plants–persist, and most AI applications remain at laboratory or pilot scale. To move beyond generic recommendations, future work must focus on five SCB-specific priorities: (i) Benchmarking ML model performance across different SCB varieties and pretreatment histories, using standardized datasets to understand generalizability. (ii) Developing transfer learning approaches that adapt laboratory-trained models to industrial conditions with limited data, bridging the lab-to-fab gap. (iii) Creating standardized SCB reference materials (with known composition and pretreatment history) to enable fair, cross-study ML model validation. (iv) Embedding AI into full-scale industrial control systems with real-time adaptability, explainability, and fault tolerance under sensor drift, missing data, and feedstock variability. (v) Fostering policy frameworks that incentivize circular bioeconomy transitions while protecting traditional biomass uses and ensuring equitable benefit sharing with local communities. Closing these gaps will position SCB as a global model for converting agro-industrial waste into regenerative, low-carbon industrial systems, directly supporting sustainable and intelligent agriculture.
