# Supplementary material for: Integrating artificial intelligence and conventional approaches in sugarcane bagasse biorefineries: a review towards a circular bioeconomy
Source: Front Bioeng Biotechnol. 2026 Jun 17;14:1879932. doi: 10.3389/fbioe.2026.1879932 (PMC13338604; doi:10.3389/fbioe.2026.1879932)
Supplement: Supplementary file 1 [file Table1.docx]

**Supplementary files:**

**Table S1:** Advantages and disadvantages of physical, chemical, and biological pretreatment strategies commonly used for SCB.

| **Pretreatments** | **Advantages of pretreatment methods** | **References** |
| --- | --- | --- |
| **1. Physical pretreatments** |  |  |
| 1. **Milling** | Alterations in the structure. | (Da Silva et al., 2010) |
| 1. **Electromagnetic waves** | The process offers benefits such as reduced reaction temperature, quicker response time, energy efficiency, increased yield, reduced chemicals and solvents, and reduced byproduct production. | (Dulie et al., 2021) |
| 1. **Ultrasonication** | Cellulose and hemicellulose have a strong effect on lignin removal, leading to increased yield and brittleness of the pretreated material. | (Ramadoss and Muthukumar, 2014) |
| **2. Chemical pretreatments** | Chemical reactions occur more quickly and efficiently because of the reduction in reaction time. | (Haldar and Purkait, 2022) |
| 1. **Persulfate** | It is an oxidizing agent that has great potential for breaking down organic contaminants. | (Miklos et al., 2018) |
| 1. **Dilute acid** | The procedure is cost-effective and can be performed under less severe conditions by reducing hemicellulose content and increasing cellulose exposure for enzymatic hydrolysis. | (Kumar et al., 2021) |
| 1. **Acid-functionalized magnetic nanoparticles** | Nanotechnology-based methods are more effective than conventional acid pretreatment in facilitating high-volume xylose liberation from SCB, offering a targeted, quick, and affordable solution for sugar mobilization from lignocellulosic materials. | (Ingle et al., 2020) |
| 1. **Alkaline pretreatment** | Alkaline pretreatment is cost-effective under milder reaction conditions and is ideal for bagasse delignification. It removes uronic acid substitutions and acetyl groups, thereby increasing cellulose accessibility. This method, using sodium hydroxide, calcium hydroxide, and ammonia, is suitable for cellulose separation and delignification in SCB. | (Konde et al., 2021) |
| 1. **Organosolv** | This process is beneficial for separating bagasse into cellulose, lignin, and hemicellulose, and optimizing solvent recovery and reuse. It reduces biomass recalcitrance and increases the enzymatic availability of carbohydrates. Low-boiling-point alcohols are often used as organo-solvents. | (Sidiras et al., 2022) |
| **3. Physicochemical Pretreatment** | | |
| 1. **Steam explosion** | This environmentally friendly pretreatment method is ideal for SCB pretreatment because of its cost-effectiveness, scalability, and reduced chemical risks. It achieves sugar release quickly and economically, and offers a wide range of applications. | (Barciela et al., 2023) |
| **4. Biological pretreatment** | By using enzymatic or microbial delignification, this environmentally friendly process greatly increases the accessibility of polysaccharides. | (Tsegaye et al., 2019) |
|  | Pretreatments break down complex biomass networks, increasing fermentation and saccharification efficiency and improving biobased product recovery. | (Liguori and Faraco, 2016) |
|  | Biological pretreatment is generally preferred over physical and chemical pretreatments because it uses less energy and produces fewer inhibitors. | (Malhotra and Suman, 2021) |
|  | Biological pretreatment of lignocellulosic biomass is facilitated by the addition of biological components, such as microorganisms or enzymes. | (Vu et al., 2020) |
|  | Microorganisms such as bacteria and fungi are used because of their metabolic capacity to convert lignocellulosic biomass into valuable products through microbial extracellular enzyme production. | (Silva et al., 2018) |
|  | Enzymatic hydrolysis efficiently converts cellulose and hemicellulose polymers into simple sugars without hazardous byproducts and under milder environmental conditions. | (Sharma et al., 2020) |
| **Disadvantages of pretreatment methods** | | |
| 1. **Physical pretreatments** | | |
| 1. **Milling** | High energy requirements. | (Da Silva et al., 2010) |
| 1. **Ultrasonication** | It does not immediately cause cellulose to saccharify into sugars. | (Ramadoss and Muthukumar, 2014) |
| 1. **Chemical pretreatments** | However, its poor selectivity may cause unintended parallel reactions. | (Sharma et al., 2020) |
| 1. **Dilute acid** | Furfural, produced from pentoses or hexoses, can cause fermentation sugar loss due to acid hydrolysis, but this can be avoided with organic acids such as acetic or citric acid. | (Kumar et al., 2021) |
| 1. **Organosolv** | Because it requires specialized instruments to function at high pressures, it is expensive. | (Santo et al., 2018) |
| 1. **Biological methods** | The biological conversion of biomass is challenging because of recalcitrance of SCB and limited lignin breakdown, resulting in prolonged hydrolysis periods. | (Sarker et al., 2021) |

**Table S2:** List of some bioproducts obtained by utilizing SCB as the substrate.

| **Products** | **Substrates** | **Pretreatment technique** | **Characterization of SCB chemical composition after pretreatment/ or its products** | **Microorganisms used/Techniques** | **References** |
| --- | --- | --- | --- | --- | --- |
| 1. **Polymeric composites** |  |  |  |  |  |
| 1. **Nanocellulose** | SCB | Chemo-mechanical method, 15 wt% NaOH solution, 1 mol HCL solution, 2 wt% NaOH solution, high-speed grinder at 1500 rpm. | - | Isolation of the nanocellulose of SCB | (Gond et al., 2021) |
| 1. **Nanofibrillated cellulose films** | SCB | Acidic natural deep eutectic solvents synergistically with acetosolv | Scanning electron microscopic (SEM), transmission electron microscopic (TEM) and Dynamic light scattering (DLS) | Using green approach | (Pandey et al., 2024) |
| 1. **Cellulose nanocrystals (CNC)** | Untreated and pretreated SCB | Enzymatic hydrolysis | Atomic force microscopy (AFM) | *Aspergillus fumigatus* CCT 7873 utilizing semi-solid fermentation | (de Oliveira Junior et al., 2020) |
| 1. **Polyvinyl alcohol (PVA)/starch/cellulose nanofiber composite film** | SCB | Ultrasonication and alkaline and mild acid treatment | Thermal characteristics, tensile strength, and elongation |  | (Ali et al., 2022) |
| 1. **Polyhydroxybutyrate (PHB)/SCB fiber composite** | *Curcuma longa* L chloroform extract and natural fibre SCB. | - | Termogravimetric analysis (TGA) and differential scanning colorimetry (DSC) measurements, and the tensile modulus |  | (Sabapathy et al., 2021) |
| 1. **Nanocellulose/nisin hybrid films** | Nisin and SCB | Mechanically ground for 11 passes | Mechanical, barrier, and optical properties |  | (Yang et al., 2020) |
| 1. **Wood plastic composites** | Solid residuals (tropical maize bagasse, sweet sorghum bagasse, and SCB) | WPC prepared by a co-rotating twin-screw extruder | Low density, strong mechanical and thermal insulation capabilities, and availability |  | (Chen et al., 2020) |
| 1. **Zinc oxide nanoparticles–cellulose composite** | SCB-extracted cellulose | SCB was ground and washed with distilled water to eliminate contaminants and impurities. This biomass was immersed in a 3.5% NaCl solution for 24 h at 60 °C, treated with 4% (w/v) NaOH for 2 h at 90 °C and washed several times. The bleaching process was carried out using 7.5 M H_2_O_2_ and 2.5 M NaOH for 24 h at 60 °C. | Fourier transform infrared, X-ray diffraction patterns, SEM, and Energy dispersive spectroscopy, thermogravimetric analysis | Fungus *Phanerochaete chrysosporium* | (Sharma et al., 2021) |
| 1. **Second-generation biofuels** | |  |  |  |  |
| **a. Bioethanol Production** | SCB and molasses | SCB was delignified using 1:10 (w/v) ratio of potassium hydroxide | SEM and  gas chromatography (GC) | Immobilized *Saccharomyces cerevisiae* SC90 | (Sowatad and Todhanakasem, 2020) |
|  | SCB | Cellulolytic enzymes |  | *Bacillus subtilis* | (Malik et al., 2021) |
|  | SCB | Dilute sulphuric acid |  | *Barnettozyma californica* | (Nouri et al., 2020) |
| **b. Biogas production** | Cow manure and SCB | Alkaline pretreatment and untreated at two different temperatures | FE-SEM | Co-digestion | (Kaur et al., 2020) |
|  | SCB | Hydrothermal pretreatment  followed by Organosolv pretreatment  during SCB thermal pretreatment and recondensed | HPLC, GC | Mixtures of microorganisms present in upflow anaerobic sludge blanket reactor (UASB) | (Lima et al., 2018) |
| 1. **Biodiesel** | SCB | 0.5 % (v/v) sulfuric acid (H_2_SO_4_) and deionized (DI) water under autoclave at 121 °C for 15 min | HPLC,  GC | *Scenedesmus acutus* | (Rattanapoltee and Kaewkannetra, 2014) |
| 1. **Biohydrogen production** | SCB | Bagasse did not require pretreatment. | SEM micrographs | *Eisenia foetida* lixiviated earthworm *vermihumus*-associated bacteria | (Oceguera-Contreras et al., 2019) |
|  | Sewage sludge (SS) with pretreated SCB | Raw and perchloric acid-pretreated SCB | HPLC,  FT-IR, Electrical conductivity | A microbial consortium | (Bu et al., 2021) |
|  | SCB | NaOH | GC,  HPLC | *Clostridium thermocellum* | (Tian et al., 2015) |
| 1. **Acids production** |  |  |  |  |  |
| 1. **Succinic acid (SA) production** | SCB | Ultrasonic pretreatment and acid hydrolysis of SCB | HPLC | *Actinobacillus succinogenes* | (Xi et al., 2013) |
|  | SCB | Hot water pretreatment of SCB, followed by high-solids enzymatic hydrolysis of pretreated residual that without washing | SEM,  FTIR,  X-ray diffractometer | *Actinobacillus* *succinogenes* ATCC 55,618 | (Xu et al., 2023) |
| **a.Lactic acid production** | SCB | Dilute acid pretreatment using 0.5% (v/v) of sulfuric acid, | HPLC | *Lactobacillus plantarum* | (de Oliveira et al., 2018) |
| 1. **Adipic acid (AA)** | SCB | Choline chloride (ChCl) was mixed with thiourea (TH) mixed with NaOH solution to form NaOH/ ChCl:TH solution | FTIR, XRD, SEM, TEM and Laser Scanning Confocal Microscope (LSCM) | *E. coli* MG1655 K12 | (Wu et al., 2023) |
| 1. **Bioelectricity** | SCB | Mixture of dry bagasse and distilled water at a ratio of 1:10, to produce sugarcane bagasse extract | MFC,  SEM | *Saccharomyces cerevisiae* | (Christwardana et al., 2021) |
| 1. **Xylooligosaccharides** | Raw and steam-exploded SCB | Hydrothermal, organosolv and alkaline for raw bagasse, and hot water extraction of the steam-exploded SCB | HPLC | endoxylanase | (Milessi et al., 2021) |
|  | Steam-exploded SCB | Thermochemical and biological processes | Capillary electrophoresis was used to quantify the samples using high-performance anion exchange chromatography. | *Clostridium thermocellum* ATCC 27405 | (Mandelli et al., 2014) |
| 1. **Food additive** | SCB | 10 mL of 90% methanol | Liquid Chromatography-Mass Spectrometry | - | (Velazquez-Martinez et al., 2021) |
| 1. **Dietary cookies** | SCB wastes, incorporated into wheat flour | Alkaline treatment using hydrogen peroxide (AHP) and sodium hydroxide | FTIR, X-ray diffraction (XRD), SEM |  | (Licona-Aguilar et al., 2023) |
| 1. **β-Carotene** | SCB | NaOH to remove lignin |  | *Rhodotorula glutinis* CCT-2186 | (D\’\iaz-Ruiz et al., 2023) |
| 1. **Composting** | SCB | Untreated SCB (UT-SCB), chemically processed SCB 1% NaOH (AT-SCB), 1:10 Methyltrioctylammonium chloride along with ethanol and alkali | FT-IR | *Bacillus aestuarii* UE25 | (Ansari et al., 2021) |
| 1. **Biofertilizers** | SCB | Solid-state fermentation |  | *Aspergillus niger, Penicillium canescens, Eupenicillium ludwigii,* and *Penicillium islandicum* | (Mendes et al., 2013) |
|  | SCB | Mechanical-biological approach | XRD, X-ray fluorescence, field emission scanning electron microscopy, and total surface area measurements | *Aspergillus niger* | (Klaic et al., 2017) |
| 1. **Cellulose production** | SCB | Acid hydrolysis | - | *Komagateibacter xylinus* | (Lin et al., 2022) |
| **12. Enzymes production** |  |  |  |  |  |
| 1. **Phosphatases Enzyme** | SCB | Biological, alkali and IL | SEM with EDX-, FTIR-, and NMR | *Neobacillus sedimentimangrovi* UE25, *Pseudomonas* species | (Ejaz et al., 2024) |
| 1. **Pectinase enzyme** | Wheat bran and SCB | - | - | *Aspergillus niger* | (Suresh and Viruthagiri, 2010) |
|  |  |  |  |  |  |
| 1. **Inulinase enzyme** | SCB and corn steep liquor | Solid-state fermentation |  | *Kluyveromyces marxianus* NRRL Y-7571 | (Mazutti et al., 2006) |
| **13. Production of mixed products** |  |  |  |  |  |
| 1. **Production of H_2_ and organic acids** | SCB | Biological treatment |  | *Chaetomium cupreum* | (Dionizio et al., 2022) |
| 1. **Production of ethanol, xylitol, and single cell protein** | SCB | Biological treatment enzymatic hydrolysis |  | *Spathaspora passalidarum* | (Bonan et al., 2021) |
| 1. **Bioethanol and biogas production** | SCB | Undergone hydrothermal processing and alkaline extraction (AE) and advanced oxidative pretreatment (AOP) | GC, HPLC, SEM | *S. cerevisiae* | (Bittencourt et al., 2019) |
| 1. **Product biorefineries** | SCB and waste in biorefineries attached to already-existing sugar mills | Combined heat and power plant |  | *Candida oleophila* ATCC 20177 and *Brevibacterium divaricatum* NRRL 8–231 | (Özüdo\ugru et al., 2019) |
| 1. **Production of hydrogen and acetone-butanol-ethanol** | SCB and rice straw | Alkaline-pretreated SCB | GC, HPLC, SEM. | Co-culture of *Enterobacter aerogenes* and *Clostridium acetobutylicum* | (Tondro et al., 2020) |
| 1. **Cellulose and cellulase production** | SCB | Alkali or with methyltrioctylammonium chloride as new IL | SEM, | *Aneurinibacillus thermoaerophilus* | (Ejaz et al., 2020)‏ |
| 1. **Production of xylanases, XOS and lignocellulose** | SCB | KOH or H_2_O_2_ | HPLC | *Aspergillus fumigatus* CCT7732 | (Carvalho et al., 2020) |
| 1. **Prebiotic XOS, cellulosic ethanol, cellulose nanofibrils and lignin nanoparticles** | sugarcane bagasse | Continuous steam explosion pilot unit,  alkaline treatment/acid precipitation | HPLC, |  | (Pereira et al., 2021) |
| 1. **Biofuels production** |  | Hydrothermal (HT) pretreatment | XRD, GC, HPLC | *Clostridium, Acetomicrobium,* and *Methanoculleus* | (Soares et al., 2021) |
| 1. **Enzymatic cocktail** | SCB | Alkali-pretreated SCB | SEM, FTIR and gravimetric analysis | *Bacillus vallismortis MH 1* and *Bacillus aestuarii UE25* | (Ejaz et al., 2021) |
|  | SCB | IL and alkali-pretreated | NMR, FTIR and SEM | *Aneurinibacillus thermoaerophilus* and *Brevibacillus borstelensis* | (Rashid et al., 2020) |
|  | SCB | Hydrothermally pretreated bagasse | HPLC | *Trichoderma reesei* and *genetically modified Escherichia coli* | (Bussamra et al., 2015) |
|  | SCB and brewery spent grain | Three pretreatments were used: alkaline, boiling water, and autoclave. | SEM, HPLC | *Aspergillus niger* | (Moran-Aguilar et al., 2021) |

**Table S3.**

 Documented industrial deployments of AI and advanced control systems in sugarcane processing facilities.

| **Facility / Company** | **Technology / System** | **Key quantified outcomes** | **Reference** |
| --- | --- | --- | --- |
| **bp bioenergy, Brazil** | S‑PAA real‑time optimization (RTO) based on AI/ML | ↑ 0.13% extraction productivity; ↓ 1.6% steam consumption; ↓ 1.1% bagasse consumption | bp bioenergy (2025) |
| **Usina São Manoel, Brazil** | MM.IA intelligent milling control (NIR + AI) | ↑ 0.25% sugar recovery; ~60,000 USD additional revenue/season | Usina São Manoel (2025) |
| **Maharashtra mill, India** | AI Co‑pilot (Findability Sciences) | ↑ 0.5–1.2% sugar recovery; ↓ 5–15% steam consumption | Mahurkar (2026) |
| **Cenicaña, Colombia** | DataCane (Industry 4.0 platform with AI/analytics) | ↓ downtime; ↑ operational efficiency; optimised predictive maintenance | Calpa Pantoja (2025) |
| **Cenicaña, Colombia** | CeniCristal (AI‑powered digital image processing for crystal monitoring) | <3% error; ↓ 5–12% crystal CV; deployed in 8 mills; early fault detection | Hernández et al. (2025) |

**Note:** TRL based on common definitions: 1–3 basic research, 4–5 laboratory validation, 6–7 pilot/demonstration, 8–9 industrial deployment. The examples above correspond to TRL 8–9 (operational in real industrial environments).

**References**

Ali, M. A. S. S., Jimat, D. N., Nawawi, W. M. F. W., and Sulaiman, S. (2022). Antibacterial, mechanical and thermal properties of PVA/starch composite film reinforced with cellulose nanofiber of sugarcane bagasse. *Arab. J. Sci. Eng.*, 1–8.

Ansari, M., Zafar, U., Ejaz, U., Sohail, M., Pirzada, A., and Aman, A. (2021). Comparison of composting of chemically pretreated and fermented sugarcane bagasse for zero-waste biorefinery. *J. Mater. Cycles Waste Manag.* 23, 911–921.

Barciela, P., Perez-Vazquez, A., Fraga-Corral, M., and Prieto, M. A. (2023). Utility aspects of sugarcane bagasse as a feedstock for bioethanol production: leading role of steam explosion as a pretreatment technique. *Processes* 11, 3116.

Bittencourt, G. A., da Silva Barreto, E., Brandão, R. L., Baêta, B. E. L., and Gurgel, L. V. A. (2019). Fractionation of sugarcane bagasse using hydrothermal and advanced oxidative pretreatments for bioethanol and biogas production in lignocellulose biorefineries. *Bioresour. Technol.* 292, 121963.

Bonan, C. I. D. G., Tramontina, R., dos Santos, M. W., Biazi, L. E., Soares, L. B., Pereira, I. O., et al. (2021). Biorefinery platform for Spathaspora passalidarum NRRL Y-27907 in the production of ethanol, xylitol, and single cell protein from sugarcane bagasse. *BioEnergy Res.*, 1–13.

Bu, J., Wei, H.-L., Wang, Y.-T., Cheng, J.-R., and Zhu, M.-J. (2021). Biochar boosts dark fermentative H2 production from sugarcane bagasse by selective enrichment/colonization of functional bacteria and enhancing extracellular electron transfer. *Water Res.* 202, 117440.

Bussamra, B. C., Freitas, S., and da Costa, A. C. (2015). Improvement on sugar cane bagasse hydrolysis using enzymatic mixture designed cocktail. *Bioresour. Technol.* 187, 173–181.

Carvalho, A. F. A., de Figueiredo, F. C., Campioni, T. S., Pastore, G. M., and de Oliva Neto, P. (2020). Improvement of some chemical and biological methods for the efficient production of xylanases, xylooligosaccharides and lignocellulose from sugar cane bagasse. *Biomass and Bioenergy* 143, 105851.

Chen, B., Luo, Z., Chen, H., Chen, C., Cai, D., Qin, P., et al. (2020). Wood plastic composites from the waste lignocellulosic biomass fibers of bio-fuels processes: a comparative study on mechanical properties and weathering effects. *Waste and Biomass Valorization* 11, 1701–1710.

Christwardana, M., Joelianingsih, J., and Yoshi, L. A. (2021). Performance of yeast microbial fuel cell integrated with sugarcane bagasse fermentation for cod reduction and electricity generation. *Bull. Chem. React. Eng. \& Catal.* 16, 446–458.

D\’\iaz-Ruiz, E., Balbino, T. R., Dos Santos, J. C., Kumar, V., da Silva, S. S., and Chandel, A. K. (2023). Fermentative Production of $β$-Carotene from Sugarcane Bagasse Hydrolysate by Rhodotorula glutinis CCT-2186. *Appl. Biochem. Biotechnol.*, 1–17.

Da Silva, A. S., Inoue, H., Endo, T., Yano, S., and Bon, E. P. S. (2010). Milling pretreatment of sugarcane bagasse and straw for enzymatic hydrolysis and ethanol fermentation. *Bioresour. Technol.* 101, 7402–7409.

de Oliveira Junior, S. D., Asevedo, E. A., de Araujo, J. S., Brito, P. B., dos Santos Cruz Costa, C. L., de Macedo, G. R., et al. (2020). Enzymatic extract of Aspergillus fumigatus CCT 7873 for hydrolysis of sugarcane bagasse and generation of cellulose nanocrystals (CNC). *Biomass Convers. Biorefinery*, 1–12.

de Oliveira, R. A., Rossell, C. E. V., Venus, J., Rabelo, S. C., and Maciel Filho, R. (2018). Detoxification of sugarcane-derived hemicellulosic hydrolysate using a lactic acid producing strain. *J. Biotechnol.* 278, 56–63.

Dionizio, B. S., Rabelo, C. A. B. S., de Jesus, H. C. R., Varesche, M. B. A., and de Souza, D. H. F. (2022). The deconstruction of the lignocellulolytic structure of sugarcane bagasse by laccases improves the production of H2 and organic acids. *Appl. Biochem. Biotechnol.* 194, 3145–3166.

Dulie, N. W., Woldeyes, B., Demsash, H. D., and Jabasingh, A. S. (2021). An insight into the valorization of hemicellulose fraction of biomass into furfural: catalytic conversion and product separation. *Waste and Biomass Valorization* 12, 531–552.

Ejaz, U., Muhammad, S., Hashmi, I. A., Ali, F. I., and Sohail, M. (2020). Utilization of methyltrioctylammonium chloride as new ionic liquid in pretreatment of sugarcane bagasse for production of cellulase by novel thermophilic bacteria. *J. Biotechnol.* 317, 34–38.

Ejaz, U., Shazad, Y., Hassan, M., and Sohail, M. (2021). Statistical optimization of saccharificaion of carbohydrate content of alkali pretreated sugarcane bagasse by enzyme cocktail produced by Bacillus vallismortis MH 1 and Bacillus aestuarii UE25. *Carbohydr. Polym. Technol. Appl.* 2, 100174.

Ejaz, U., Sohail, M., El-Bahy, Z. M., Salem, M. A., and Alzahrani, A. Y. (2024). Utilization of hydrolysate from saccharified sugarcane bagasse for phosphatases production. *Biomass Convers. Biorefinery* 14, 5331–5342.

Gond, R. K., Gupta, M. K., and Jawaid, M. (2021). Extraction of nanocellulose from sugarcane bagasse and its characterization for potential applications. *Polym. Compos.* 42, 5400–5412.

Haldar, D., and Purkait, M. K. (2022). Thermochemical pretreatment enhanced bioconversion of elephant grass (Pennisetum purpureum): insight on the production of sugars and lignin. *Biomass Convers. Biorefinery*, 1–14.

Ingle, A. P., Philippini, R. R., and da Silva, S. S. (2020). Pretreatment of sugarcane bagasse using two different acid-functionalized magnetic nanoparticles: a novel approach for high sugar recovery. *Renew. Energy* 150, 957–964.

Kaur, M., Verma, Y. P., Chauhan, S., and others (2020). Effect of chemical pretreatment of sugarcane bagasse on biogas production. *Mater. Today Proc.* 21, 1937–1942.

Klaic, R., Plotegher, F., Ribeiro, C., Zangirolami, T. C., and Farinas, C. S. (2017). A novel combined mechanical-biological approach to improve rock phosphate solubilization. *Int. J. Miner. Process.* 161, 50–58.

Konde, K. S., Nagarajan, S., Kumar, V., Patil, S. V, and Ranade, V. V (2021). Sugarcane bagasse based biorefineries in India: potential and challenges. *Sustain. Energy \& Fuels* 5, 52–78.

Kumar, A., Kumar, V., Singh, B., and others (2021). Cellulosic and hemicellulosic fractions of sugarcane bagasse: Potential, challenges and future perspective. *Int. J. Biol. Macromol.* 169, 564–582.

Licona-Aguilar, Á. I., Lois-Correa, J. A., Torres-Huerta, A. M., Dom\’\inguez-Crespo, M. A., Urdapilleta-Inchaurregui, V., Rodr\’\iguez-Salazar, A. E., et al. (2023). Production of dietary cookies based on wheat-sugarcane bagasse: Determination of textural, proximal, sensory, physical and microbial parameters. *LWT* 184, 115061.

Liguori, R., and Faraco, V. (2016). Biological processes for advancing lignocellulosic waste biorefinery by advocating circular economy. *Bioresour. Technol.* 215, 13–20.

Lima, D. R. S., Adarme, O. F. H., Baêta, B. E. L., Gurgel, L. V. A., and de Aquino, S. F. (2018). Influence of different thermal pretreatments and inoculum selection on the biomethanation of sugarcane bagasse by solid-state anaerobic digestion: a kinetic analysis. *Ind. Crops Prod.* 111, 684–693.

Lin, S.-P., Huang, S.-H., Ting, Y., Hsu, H.-Y., and Cheng, K.-C. (2022). Evaluation of detoxified sugarcane bagasse hydrolysate by atmospheric cold plasma for bacterial cellulose production. *Int. J. Biol. Macromol.* 204, 136–143.

Malhotra, M., and Suman, S. K. (2021). Laccase-mediated delignification and detoxification of lignocellulosic biomass: removing obstacles in energy generation. *Environ. Sci. Pollut. Res.* 28, 58929–58944.

Malik, W. A., Khan, H. M., and Javed, S. (2021). Bioprocess optimization for enhanced production of bacterial cellulase and hydrolysis of sugarcane bagasse. *BioEnergy Res.*, 1–14.

Mandelli, F., Brenelli, L. B., Almeida, R. F., Goldbeck, R., Wolf, L. D., Hoffmam, Z. B., et al. (2014). Simultaneous production of xylooligosaccharides and antioxidant compounds from sugarcane bagasse via enzymatic hydrolysis. *Ind. Crops Prod.* 52, 770–775.

Mazutti, M., Bender, J. P., Treichel, H., and Di Luccio, M. (2006). Optimization of inulinase production by solid-state fermentation using sugarcane bagasse as substrate. *Enzyme Microb. Technol.* 39, 56–59.

Mendes, G. O., Dias, C. S., Silva, I. R., Júnior, J. I. R., Pereira, O. L., and Costa, M. D. (2013). Fungal rock phosphate solubilization using sugarcane bagasse. *World J. Microbiol. Biotechnol.* 29, 43–50.

Miklos, D. B., Remy, C., Jekel, M., Linden, K. G., Drewes, J. E., and Hübner, U. (2018). Evaluation of advanced oxidation processes for water and wastewater treatment--A critical review. *Water Res.* 139, 118–131.

Milessi, T. S., Corradini, F. A. S., Marçal, J. V. M., Baldez, T. O., Kopp, W., Giordano, R. C., et al. (2021). Xylooligosaccharides production chain in sugarcane biorefineries: From the selection of pretreatment conditions to the evaluation of nutritional properties. *Ind. Crops Prod.* 172, 114056.

Moran-Aguilar, M. G., Costa-Trigo, I., Calderón-Santoyo, M., Dom\’\inguez, J. M., and Aguilar-Uscanga, M. G. (2021). Production of cellulases and xylanases in solid-state fermentation by different strains of Aspergillus niger using sugarcane bagasse and brewery spent grain. *Biochem. Eng. J.* 172, 108060.

Nouri, H., Ahi, M., Azin, M., and Gargari, S. L. M. (2020). Detoxification vs. adaptation to inhibitory substances in the production of bioethanol from sugarcane bagasse hydrolysate: A case study. *Biomass and Bioenergy* 139, 105629.

Oceguera-Contreras, E., Aguilar-Juárez, O., Oseguera-Galindo, D., Mac\’\ias-Barragán, J., Bolanos-Rosales, R., Mena-Enr\’\iquez, M., et al. (2019). Biohydrogen production by vermihumus-associated microorganisms using agro industrial wastes as substrate. *Int. J. Hydrogen Energy* 44, 9856–9865.

Özüdo\ugru, H. M. R., Nieder-Heitmann, M., Haigh, K. F., and Görgens, J. F. (2019). Techno-economic analysis of product biorefineries utilizing sugarcane lignocelluloses: Xylitol, citric acid and glutamic acid scenarios annexed to sugar mills with electricity co-production. *Ind. Crops Prod.* 133, 259–268.

Pandey, A., Kalamdhad, A. S., and Sharma, Y. C. (2024). Sustainable upcycling of sugarcane bagasse into nanofibrillated cellulose utilizing novel green solvents and high intensity ultrasonication. *Sustain. Chem. Pharm.* 37, 101373.

Pereira, B., Marcondes, W. F., Carvalho, W., and Arantes, V. (2021). High yield biorefinery products from sugarcane bagasse: Prebiotic xylooligosaccharides, cellulosic ethanol, cellulose nanofibrils and lignin nanoparticles. *Bioresour. Technol.* 342, 125970.

Ramadoss, G., and Muthukumar, K. (2014). Ultrasound assisted ammonia pretreatment of sugarcane bagasse for fermentable sugar production. *Biochem. Eng. J.* 83, 33–41.

Rashid, R., Ejaz, U., Ali, F. I., Hashmi, I. A., Bari, A., Liu, J., et al. (2020). Combined pretreatment of sugarcane bagasse using alkali and ionic liquid to increase hemicellulose content and xylanase production. *BMC Biotechnol.* 20, 1–15.

Rattanapoltee, P., and Kaewkannetra, P. (2014). Utilization of agricultural residues of pineapple peels and sugarcane bagasse as cost-saving raw materials in Scenedesmus acutus for lipid accumulation and biodiesel production. *Appl. Biochem. Biotechnol.* 173, 1495–1510.

Sabapathy, P. C., Devaraj, S., Anburajan, P., Parvez, A., Kathirvel, P., and Qi, X. (2021). Active polyhydroxybutyrate (PHB)/sugarcane bagasse fiber-based anti-microbial green composite: material characterization and degradation studies. *Appl. Nanosci.*, 1–13.

Santo, M. E., Rezende, C. A., Bernardinelli, O. D., Pereira Jr, N., Curvelo, A. A. S., Deazevedo, E. R., et al. (2018). Structural and compositional changes in sugarcane bagasse subjected to hydrothermal and organosolv pretreatments and their impacts on enzymatic hydrolysis. *Ind. Crops Prod.* 113, 64–74.

Sarker, T. R., Pattnaik, F., Nanda, S., Dalai, A. K., Meda, V., and Naik, S. (2021). Hydrothermal pretreatment technologies for lignocellulosic biomass: A review of steam explosion and subcritical water hydrolysis. *Chemosphere* 284, 131372.

Sharma, B., Larroche, C., and Dussap, C.-G. (2020). Comprehensive assessment of 2G bioethanol production. *Bioresour. Technol.* 313, 123630.

Sharma, J. L., Dhayal, V., and Sharma, R. K. (2021). White-rot fungus mediated green synthesis of zinc oxide nanoparticles and their impregnation on cellulose to develop environmental friendly antimicrobial fibers. *3 Biotech* 11, 269.

Sidiras, D., Politi, D., Giakoumakis, G., and Salapa, I. (2022). Simulation and optimization of organosolv based lignocellulosic biomass refinery: A review. *Bioresour. Technol.* 343, 126158.

Silva, T. A. L., Zamora, H. D. Z., Varão, L. H. R., Prado, N. S., Baffi, M. A., and Pasquini, D. (2018). Effect of steam explosion pretreatment catalysed by organic acid and alkali on chemical and structural properties and enzymatic hydrolysis of sugarcane bagasse. *Waste and Biomass Valorization* 9, 2191–2201.

Soares, L. A., Silva, E. L., and Varesche, M. B. A. (2021). Dissecting the role of heterogeneity and hydrothermal pretreatment of sugarcane bagasse in metabolic pathways for biofuels production. *Ind. Crops Prod.* 160, 113120.

Sowatad, A., and Todhanakasem, T. (2020). Bioethanol production by repeated batch using immobilized yeast cells on sugarcane bagasse. *Waste and Biomass Valorization* 11, 2009–2016.

Suresh, B., and Viruthagiri, T. (2010). Optimization and kinetics of pectinase enzyme using Aspergillus niger by solid-state fermentation. *Indian J. Sci. Technol.* 3, 867–870.

Tian, Q.-Q., Liang, L., and Zhu, M.-J. (2015). Enhanced biohydrogen production from sugarcane bagasse by Clostridium thermocellum supplemented with CaCO3. *Bioresour. Technol.* 197, 422–428.

Tondro, H., Musivand, S., Zilouei, H., Bazarganipour, M., and Zargoosh, K. (2020). Biological production of hydrogen and acetone-butanol-ethanol from sugarcane bagasse and rice straw using co-culture of Enterobacter aerogenes and Clostridium acetobutylicum. *Biomass and Bioenergy* 142, 105818.

Tsegaye, B., Balomajumder, C., and Roy, P. (2019). Microbial delignification and hydrolysis of lignocellulosic biomass to enhance biofuel production: an overview and future prospect. *Bull. Natl. Res. Cent.* 43, 1–16.

Velazquez-Martinez, V., Valles-Rosales, D., Rodriguez-Uribe, L., Holguin, O., Quintero-Quiroz, J., Reyes-Jaquez, D., et al. (2021). Antimicrobial, shelf-life stability, and effect of maltodextrin and gum arabic on the encapsulation efficiency of sugarcane bagasse bioactive compounds. *Foods* 10, 116.

Vu, H. P., Nguyen, L. N., Vu, M. T., Johir, M. A. H., McLaughlan, R., and Nghiem, L. D. (2020). A comprehensive review on the framework to valorise lignocellulosic biomass as biorefinery feedstocks. *Sci. Total Environ.* 743, 140630.

Wu, M., Di, J., Gong, L., He, Y.-C., Ma, C., and Deng, Y. (2023). Enhanced adipic acid production from sugarcane bagasse by a rapid room temperature pretreatment. *Chem. Eng. J.* 452, 139320.

Xi, Y., Dai, W., Xu, R., Zhang, J., Chen, K., Jiang, M., et al. (2013). Ultrasonic pretreatment and acid hydrolysis of sugarcane bagasse for succinic acid production using Actinobacillus succinogenes. *Bioprocess Biosyst. Eng.* 36, 1779–1785.

Xu, C., Xiong, Y., Zhang, J., Li, K., Zhong, S., Huang, S., et al. (2023). Liquid hot water pretreatment combined with high-solids enzymatic hydrolysis and fed-batch fermentation for succinic acid sustainable processed from sugarcane bagasse. *Bioresour. Technol.* 369, 128389.

Yang, Y., Liu, H., Wu, M., Ma, J., and Lu, P. (2020). Bio-based antimicrobial packaging from sugarcane bagasse nanocellulose/nisin hybrid films. *Int. J. Biol. Macromol.* 161, 627–635.
